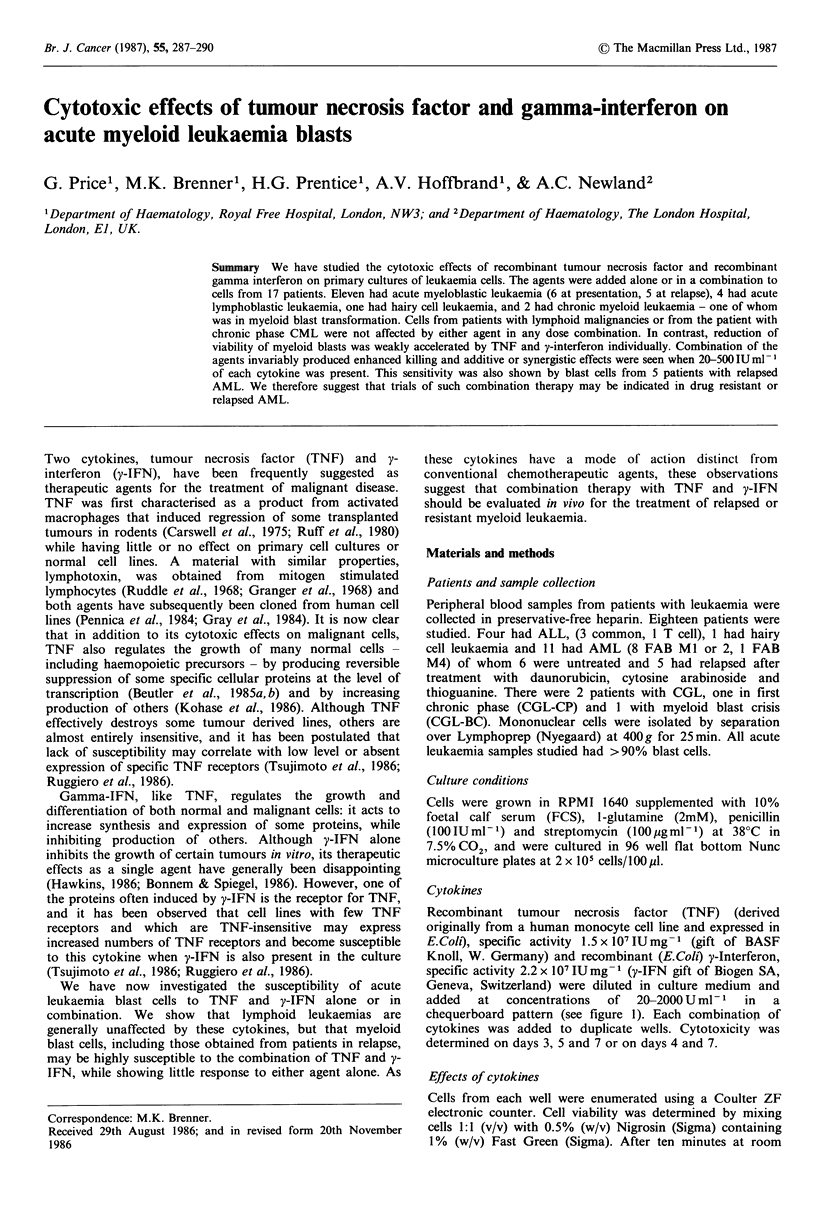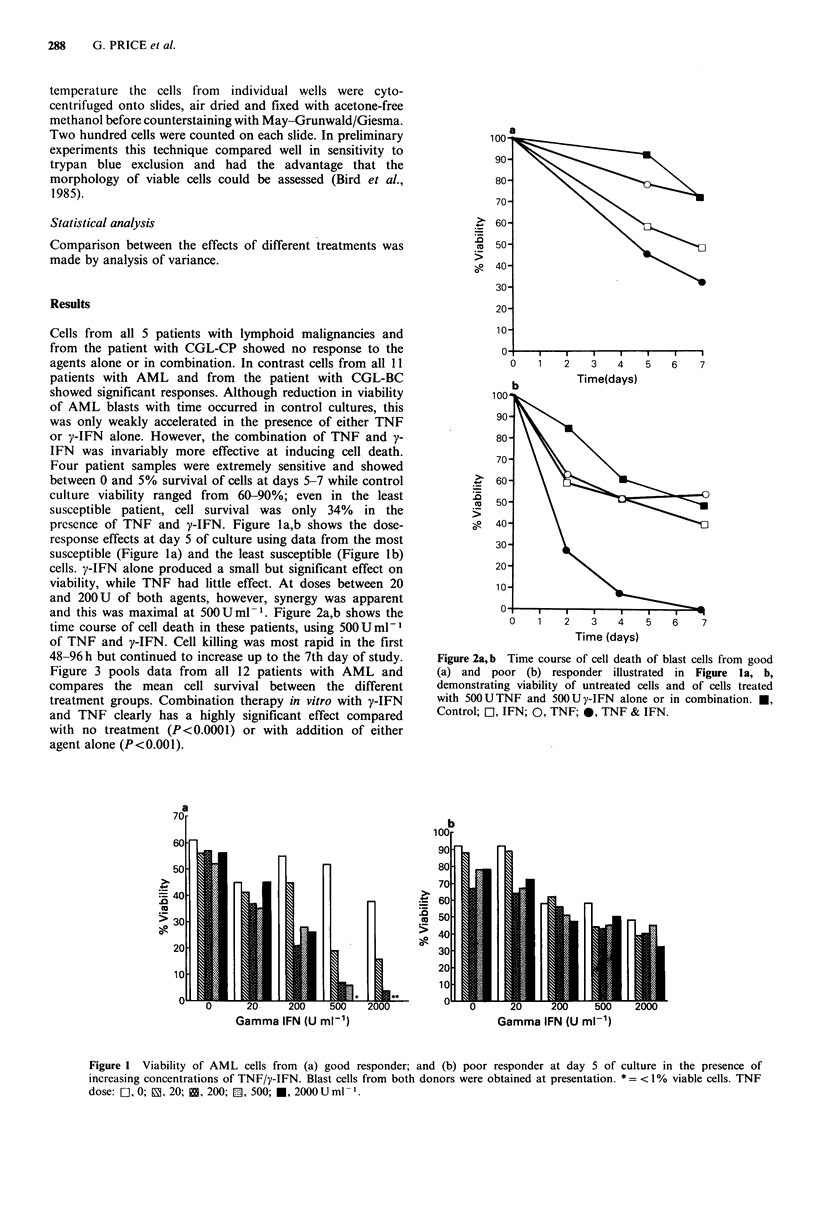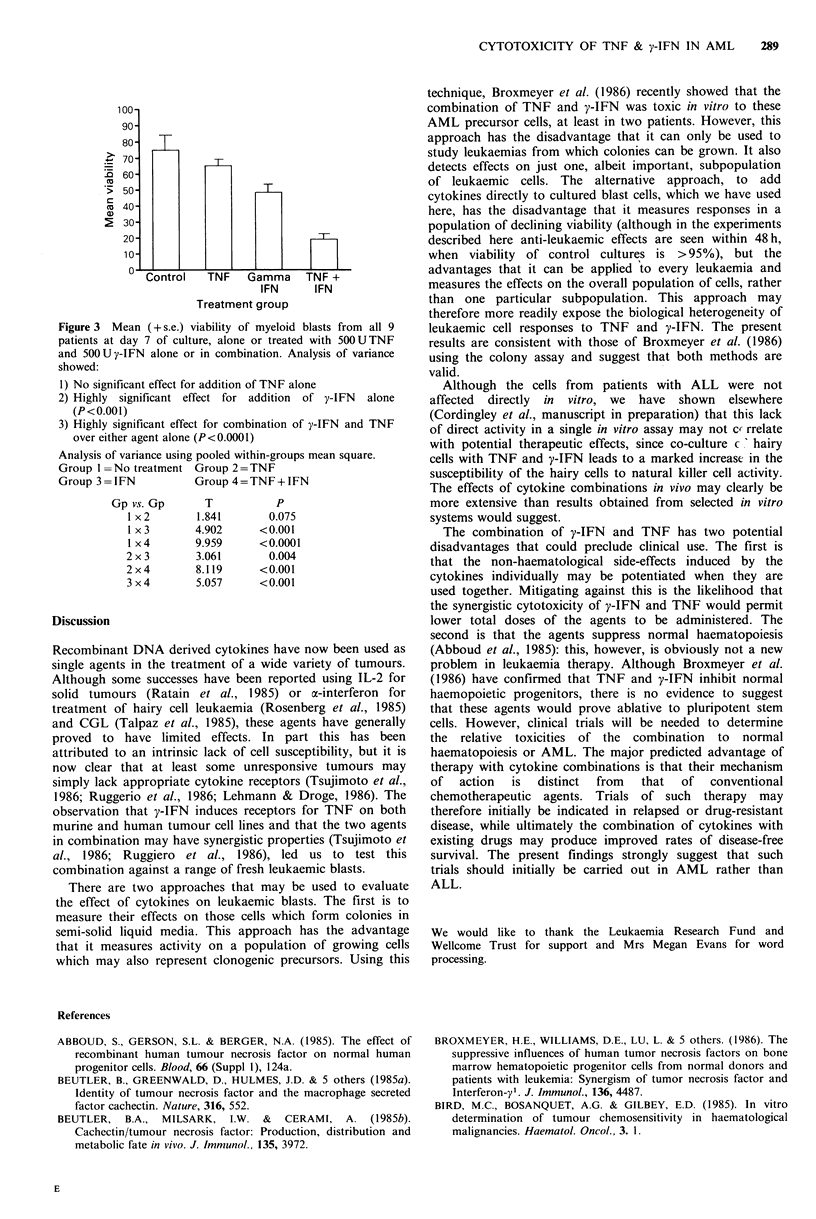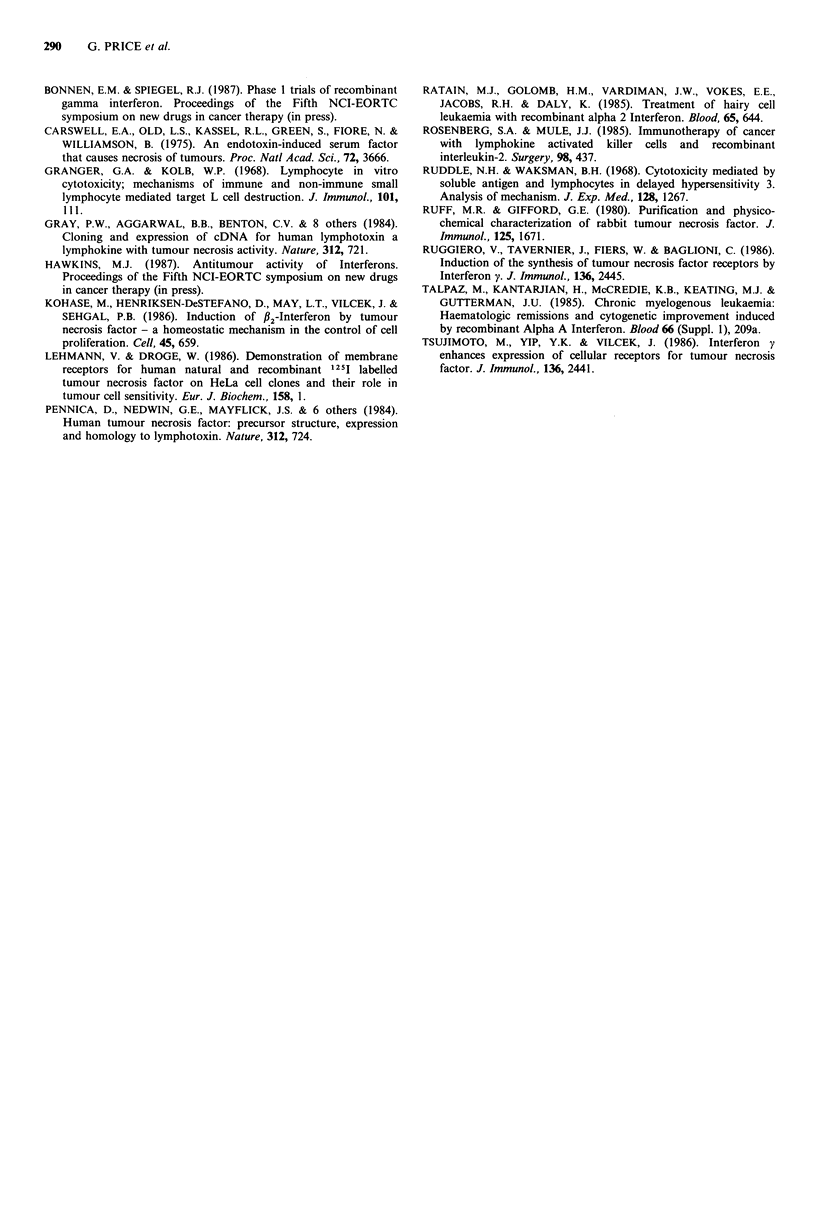# Cytotoxic effects of tumour necrosis factor and gamma-interferon on acute myeloid leukaemia blasts.

**DOI:** 10.1038/bjc.1987.55

**Published:** 1987-03

**Authors:** G. Price, M. K. Brenner, H. G. Prentice, A. V. Hoffbrand, A. C. Newland

## Abstract

We have studied the cytotoxic effects of recombinant tumour necrosis factor and recombinant gamma interferon on primary cultures of leukaemia cells. The agents were added alone or in a combination to cells from 17 patients. Eleven had acute myeloblastic leukaemia (6 at presentation, 5 at relapse), 4 had acute lymphoblastic leukaemia, one had hairy cell leukaemia, and 2 had chronic myeloid leukaemia--one of whom was in myeloid blast transformation. Cells from patients with lymphoid malignancies or from the patient with chronic phase CML were not affected by either agent in any dose combination. In contrast, reduction of viability of myeloid blasts was weakly accelerated by TNF and gamma-interferon individually. Combination of the agents invariably produced enhanced killing and additive or synergistic effects were seen when 20-500 IU ml-1 of each cytokine was present. This sensitivity was also shown by blast cells from 5 patients with relapsed AML. We therefore suggest that trials of such combination therapy may be indicated in drug resistant or relapsed AML.


Br. J. Cancer (1987), 55, 287–290 © The Macmillan Press Ltd., 1987

### Cytotoxic effects of tumour necrosis factor and gamma-interferon on acute myeloid leukaemia blasts

G. Price[1], M.K. Brenner[1], H.G. Prentice[1], A.V. Hoffbrand[1], & A.C. Newland[2]

[1]*Department of Haematology, Royal Free Hospital, London, NW3; and* [2]*Department of Haematology, The London Hospital, London, E1, UK.*

**Summary** We have studied the cytotoxic effects of recombinant tumour necrosis factor and recombinant gamma interferon on primary cultures of leukaemia cells. The agents were added alone or in a combination to cells from 17 patients. Eleven had acute myeloblastic leukaemia (6 at presentation, 5 at relapse), 4 had acute lymphoblastic leukaemia, one had hairy cell leukaemia, and 2 had chronic myeloid leukaemia – one of whom was in myeloid blast transformation. Cells from patients with lymphoid malignancies or from the patient with chronic phase CML were not affected by either agent in any dose combination. In contrast, reduction of viability of myeloid blasts was weakly accelerated by TNF and γ-interferon individually. Combination of the agents invariably produced enhanced killing and additive or synergistic effects were seen when 20–500 IU ml$^{-1}$ of each cytokine was present. This sensitivity was also shown by blast cells from 5 patients with relapsed AML. We therefore suggest that trials of such combination therapy may be indicated in drug resistant or relapsed AML.

Two cytokines, tumour necrosis factor (TNF) and γ-interferon (γ-IFN), have been frequently suggested as therapeutic agents for the treatment of malignant disease. TNF was first characterised as a product from activated macrophages that induced regression of some transplanted tumours in rodents (Carswell *et al.*, 1975; Ruff *et al.*, 1980) while having little or no effect on primary cell cultures or normal cell lines. A material with similar properties, lymphotoxin, was obtained from mitogen stimulated lymphocytes (Ruddle *et al.*, 1968; Granger *et al.*, 1968) and both agents have subsequently been cloned from human cell lines (Pennica *et al.*, 1984; Gray *et al.*, 1984). It is now clear that in addition to its cytotoxic effects on malignant cells, TNF also regulates the growth of many normal cells – including haemopoietic precursors – by producing reversible suppression of some specific cellular proteins at the level of transcription (Beutler *et al.*, 1985*a,b*) and by increasing production of others (Kohase *et al.*, 1986). Although TNF effectively destroys some tumour derived lines, others are almost entirely insensitive, and it has been postulated that lack of susceptibility may correlate with low level or absent expression of specific TNF receptors (Tsujimoto *et al.*, 1986; Ruggiero *et al.*, 1986).

Gamma-IFN, like TNF, regulates the growth and differentiation of both normal and malignant cells: it acts to increase synthesis and expression of some proteins, while inhibiting production of others. Although γ-IFN alone inhibits the growth of certain tumours *in vitro*, its therapeutic effects as a single agent have generally been disappointing (Hawkins, 1986; Bonnem & Spiegel, 1986). However, one of the proteins often induced by γ-IFN is the receptor for TNF, and it has been observed that cell lines with few TNF receptors and which are TNF-insensitive may express increased numbers of TNF receptors and become susceptible to this cytokine when γ-IFN is also present in the culture (Tsujimoto *et al.*, 1986; Ruggiero *et al.*, 1986).

We have now investigated the susceptibility of acute leukaemia blast cells to TNF and γ-IFN alone or in combination. We show that lymphoid leukaemias are generally unaffected by these cytokines, but that myeloid blast cells, including those obtained from patients in relapse, may be highly susceptible to the combination of TNF and γ-IFN, while showing little response to either agent alone. As these cytokines have a mode of action distinct from conventional chemotherapeutic agents, these observations suggest that combination therapy with TNF and γ-IFN should be evaluated *in vivo* for the treatment of relapsed or resistant myeloid leukaemia.

Correspondence: M.K. Brenner.
Received 29th August 1986; and in revised form 20th November 1986

### Materials and methods

### Patients and sample collection

Peripheral blood samples from patients with leukaemia were collected in preservative-free heparin. Eighteen patients were studied. Four had ALL, (3 common, 1 T cell), 1 had hairy cell leukaemia and 11 had AML (8 FAB M1 or 2, 1 FAB M4) of whom 6 were untreated and 5 had relapsed after treatment with daunorubicin, cytosine arabinoside and thioguanine. There were 2 patients with CGL, one in first chronic phase (CGL-CP) and 1 with myeloid blast crisis (CGL-BC). Mononuclear cells were isolated by separation over Lymphoprep (Nyegaard) at 400 *g* for 25 min. All acute leukaemia samples studied had >90% blast cells.

### Culture conditions

Cells were grown in RPMI 1640 supplemented with 10% foetal calf serum (FCS), l-glutamine (2mM), penicillin (100 IU ml$^{-1}$) and streptomycin (100 μg ml$^{-1}$) at 38°C in 7.5% $CO_2$, and were cultured in 96 well flat bottom Nunc microculture plates at $2 \times 10^5$ cells/100 μl.

### Cytokines

Recombinant tumour necrosis factor (TNF) (derived originally from a human monocyte cell line and expressed in *E.Coli*), specific activity $1.5 \times 10^7$ IU mg$^{-1}$ (gift of BASF Knoll, W. Germany) and recombinant (*E.Coli*) γ-Interferon, specific activity $2.2 \times 10^7$ IU mg$^{-1}$ (γ-IFN gift of Biogen SA, Geneva, Switzerland) were diluted in culture medium and added at concentrations of 20–2000 U ml$^{-1}$ in a chequerboard pattern (see figure 1). Each combination of cytokines was added to duplicate wells. Cytotoxicity was determined on days 3, 5 and 7 or on days 4 and 7.

### Effects of cytokines

Cells from each well were enumerated using a Coulter ZF electronic counter. Cell viability was determined by mixing cells 1:1 (v/v) with 0.5% (w/v) Nigrosin (Sigma) containing 1% (w/v) Fast Green (Sigma). After ten minutes at room temperature the cells from individual wells were cytocentrifuged onto slides, air dried and fixed with acetone-free methanol before counterstaining with May–Grunwald/Giesma. Two hundred cells were counted on each slide. In preliminary experiments this technique compared well in sensitivity to trypan blue exclusion and had the advantage that the morphology of viable cells could be assessed (Bird et al., 1985).

*Statistical analysis*

Comparison between the effects of different treatments was made by analysis of variance.

### Results

Cells from all 5 patients with lymphoid malignancies and from the patient with CGL-CP showed no response to the agents alone or in combination. In contrast cells from all 11 patients with AML and from the patient with CGL-BC showed significant responses. Although reduction in viability of AML blasts with time occurred in control cultures, this was only weakly accelerated in the presence of either TNF or γ-IFN alone. However, the combination of TNF and γ-IFN was invariably more effective at inducing cell death. Four patient samples were extremely sensitive and showed between 0 and 5% survival of cells at days 5–7 while control culture viability ranged from 60–90%; even in the least susceptible patient, cell survival was only 34% in the presence of TNF and γ-IFN. Figure 1a,b shows the dose-response effects at day 5 of culture using data from the most susceptible (Figure 1a) and the least susceptible (Figure 1b) cells. γ-IFN alone produced a small but significant effect on viability, while TNF had little effect. At doses between 20 and 200 U of both agents, however, synergy was apparent and this was maximal at 500 U ml$^{-1}$. Figure 2a,b shows the time course of cell death in these patients, using 500 U ml$^{-1}$ of TNF and γ-IFN. Cell killing was most rapid in the first 48–96 h but continued to increase up to the 7th day of study. Figure 3 pools data from all 12 patients with AML and compares the mean cell survival between the different treatment groups. Combination therapy *in vitro* with γ-IFN and TNF clearly has a highly significant effect compared with no treatment ($P<0.0001$) or with addition of either agent alone ($P<0.001$).

**Figure 2a, b** Time course of cell death of blast cells from good (a) and poor (b) responder illustrated in **Figure 1a, b**, demonstrating viability of untreated cells and of cells treated with 500 U TNF and 500 U γ-IFN alone or in combination. ■, Control; □, IFN; ○, TNF; ●, TNF & IFN.

**Figure 1** Viability of AML cells from (a) good responder; and (b) poor responder at day 5 of culture in the presence of increasing concentrations of TNF/γ-IFN. Blast cells from both donors were obtained at presentation. * = <1% viable cells. TNF dose: □, 0; ▧, 20; ▩, 200; ▤, 500; ■, 2000 U ml$^{-1}$.

**Figure 3** Mean (+s.e.) viability of myeloid blasts from all 9 patients at day 7 of culture, alone or treated with 500 U TNF and 500 U γ-IFN alone or in combination. Analysis of variance showed:

1) No significant effect for addition of TNF alone
2) Highly significant effect for addition of γ-IFN alone ($P<0.001$)
3) Highly significant effect for combination of γ-IFN and TNF over either agent alone ($P<0.0001$)

Analysis of variance using pooled within-groups mean square.
Group 1 = No treatment Group 2 = TNF
Group 3 = IFN Group 4 = TNF + IFN

| Gp *vs.* Gp | T | *P* |
|---|---|---|
| 1×2 | 1.841 | 0.075 |
| 1×3 | 4.902 | <0.001 |
| 1×4 | 9.959 | <0.0001 |
| 2×3 | 3.061 | 0.004 |
| 2×4 | 8.119 | <0.001 |
| 3×4 | 5.057 | <0.001 |

### Discussion

Recombinant DNA derived cytokines have now been used as single agents in the treatment of a wide variety of tumours. Although some successes have been reported using IL-2 for solid tumours (Ratain *et al.*, 1985) or α-interferon for treatment of hairy cell leukaemia (Rosenberg *et al.*, 1985) and CGL (Talpaz *et al.*, 1985), these agents have generally proved to have limited effects. In part this has been attributed to an intrinsic lack of cell susceptibility, but it is now clear that at least some unresponsive tumours may simply lack appropriate cytokine receptors (Tsujimoto *et al.*, 1986; Ruggerio *et al.*, 1986; Lehmann & Droge, 1986). The observation that γ-IFN induces receptors for TNF on both murine and human tumour cell lines and that the two agents in combination may have synergistic properties (Tsujimoto *et al.*, 1986; Ruggiero *et al.*, 1986), led us to test this combination against a range of fresh leukaemic blasts.

There are two approaches that may be used to evaluate the effect of cytokines on leukaemic blasts. The first is to measure their effects on those cells which form colonies in semi-solid liquid media. This approach has the advantage that it measures activity on a population of growing cells which may also represent clonogenic precursors. Using this technique, Broxmeyer *et al.* (1986) recently showed that the combination of TNF and γ-IFN was toxic *in vitro* to these AML precursor cells, at least in two patients. However, this approach has the disadvantage that it can only be used to study leukaemias from which colonies can be grown. It also detects effects on just one, albeit important, subpopulation of leukaemic cells. The alternative approach, to add cytokines directly to cultured blast cells, which we have used here, has the disadvantage that it measures responses in a population of declining viability (although in the experiments described here anti-leukaemic effects are seen within 48 h, when viability of control cultures is >95%), but the advantages that it can be applied to every leukaemia and measures the effects on the overall population of cells, rather than one particular subpopulation. This approach may therefore more readily expose the biological heterogeneity of leukaemic cell responses to TNF and γ-IFN. The present results are consistent with those of Broxmeyer *et al.* (1986) using the colony assay and suggest that both methods are valid.

Although the cells from patients with ALL were not affected directly *in vitro*, we have shown elsewhere (Cordingley *et al.*, manuscript in preparation) that this lack of direct activity in a single *in vitro* assay may not correlate with potential therapeutic effects, since co-culture of hairy cells with TNF and γ-IFN leads to a marked increase in the susceptibility of the hairy cells to natural killer cell activity. The effects of cytokine combinations *in vivo* may clearly be more extensive than results obtained from selected *in vitro* systems would suggest.

The combination of γ-IFN and TNF has two potential disadvantages that could preclude clinical use. The first is that the non-haematological side-effects induced by the cytokines individually may be potentiated when they are used together. Mitigating against this is the likelihood that the synergistic cytotoxicity of γ-IFN and TNF would permit lower total doses of the agents to be administered. The second is that the agents suppress normal haematopoiesis (Abboud *et al.*, 1985): this, however, is obviously not a new problem in leukaemia therapy. Although Broxmeyer *et al.* (1986) have confirmed that TNF and γ-IFN inhibit normal haemopoietic progenitors, there is no evidence to suggest that these agents would prove ablative to pluripotent stem cells. However, clinical trials will be needed to determine the relative toxicities of the combination to normal haematopoiesis or AML. The major predicted advantage of therapy with cytokine combinations is that their mechanism of action is distinct from that of conventional chemotherapeutic agents. Trials of such therapy may therefore initially be indicated in relapsed or drug-resistant disease, while ultimately the combination of cytokines with existing drugs may produce improved rates of disease-free survival. The present findings strongly suggest that such trials should initially be carried out in AML rather than ALL.

We would like to thank the Leukaemia Research Fund and Wellcome Trust for support and Mrs Megan Evans for word processing.

### References

ABBOUD, S., GERSON, S.L. & BERGER, N.A. (1985). The effect of recombinant human tumour necrosis factor on normal human progenitor cells. *Blood*, **66** (Suppl 1), 124a.

BEUTLER, B., GREENWALD, D., HULMES, J.D. & 5 others (1985*a*). Identity of tumour necrosis factor and the macrophage secreted factor cachectin. *Nature*, **316**, 552.

BEUTLER, B.A., MILSARK, I.W. & CERAMI, A. (1985*b*). Cachectin/tumour necrosis factor: Production, distribution and metabolic fate *in vivo*. *J. Immunol.*, **135**, 3972.

BROXMEYER, H.E., WILLIAMS, D.E., LU, L. & 5 others. (1986). The suppressive influences of human tumor necrosis factors on bone marrow hematopoietic progenitor cells from normal donors and patients with leukemia: Synergism of tumor necrosis factor and Interferon-$\gamma^1$. *J. Immunol.*, **136**, 4487.

BIRD, M.C., BOSANQUET, A.G. & GILBEY, E.D. (1985). In vitro determination of tumour chemosensitivity in haematological malignancies. *Haematol. Oncol.*, **3**, 1.

BONNEN, E.M. & SPIEGEL, R.J. (1987). Phase 1 trials of recombinant gamma interferon. Proceedings of the Fifth NCI-EORTC symposium on new drugs in cancer therapy (in press).

CARSWELL, E.A., OLD, L.S., KASSEL, R.L., GREEN, S., FIORE, N. & WILLIAMSON, B. (1975). An endotoxin-induced serum factor that causes necrosis of tumours. *Proc. Natl Acad. Sci.*, **72**, 3666.

GRANGER, G.A. & KOLB, W.P. (1968). Lymphocyte in vitro cytotoxicity; mechanisms of immune and non-immune small lymphocyte mediated target L cell destruction. *J. Immunol.*, **101**, 111.

GRAY, P.W., AGGARWAL, B.B., BENTON, C.V. & 8 others (1984). Cloning and expression of cDNA for human lymphotoxin a lymphokine with tumour necrosis activity. *Nature*, **312**, 721.

HAWKINS, M.J. (1987). Antitumour activity of Interferons. Proceedings of the Fifth NCI-EORTC symposium on new drugs in cancer therapy (in press).

KOHASE, M., HENRIKSEN-DeSTEFANO, D., MAY, L.T., VILCEK, J. & SEHGAL, P.B. (1986). Induction of $\beta_2$-Interferon by tumour necrosis factor – a homeostatic mechanism in the control of cell proliferation. *Cell*, **45**, 659.

LEHMANN, V. & DROGE, W. (1986). Demonstration of membrane receptors for human natural and recombinant $^{125}I$ labelled tumour necrosis factor on HeLa cell clones and their role in tumour cell sensitivity. *Eur. J. Biochem.*, **158**, 1.

PENNICA, D., NEDWIN, G.E., MAYFLICK, J.S. & 6 others (1984). Human tumour necrosis factor: precursor structure, expression and homology to lymphotoxin. *Nature*, **312**, 724.

RATAIN, M.J., GOLOMB, H.M., VARDIMAN, J.W., VOKES, E.E., JACOBS, R.H. & DALY, K. (1985). Treatment of hairy cell leukaemia with recombinant alpha 2 Interferon. *Blood*, **65**, 644.

ROSENBERG, S.A. & MULE, J.J. (1985). Immunotherapy of cancer with lymphokine activated killer cells and recombinant interleukin-2. *Surgery*, **98**, 437.

RUDDLE, N.H. & WAKSMAN, B.H. (1968). Cytotoxicity mediated by soluble antigen and lymphocytes in delayed hypersensitivity 3. Analysis of mechanism. *J. Exp. Med.*, **128**, 1267.

RUFF, M.R. & GIFFORD, G.E. (1980). Purification and physico-chemical characterization of rabbit tumour necrosis factor. *J. Immunol.*, **125**, 1671.

RUGGIERO, V., TAVERNIER, J., FIERS, W. & BAGLIONI, C. (1986). Induction of the synthesis of tumour necrosis factor receptors by Interferon $\gamma$. *J. Immunol.*, **136**, 2445.

TALPAZ, M., KANTARJIAN, H., McCREDIE, K.B., KEATING, M.J. & GUTTERMAN, J.U. (1985). Chronic myelogenous leukaemia: Haematologic remissions and cytogenetic improvement induced by recombinant Alpha A Interferon. *Blood* **66** (Suppl. 1), 209a.

TSUJIMOTO, M., YIP, Y.K. & VILCEK, J. (1986). Interferon $\gamma$ enhances expression of cellular receptors for tumour necrosis factor. *J. Immunol.*, **136**, 2441.